# Acetylcholine Reduces L-Type Calcium Current without Major Changes in Repolarization of Canine and Human Purkinje and Ventricular Tissue

**DOI:** 10.3390/biomedicines10112987

**Published:** 2022-11-21

**Authors:** Arie O. Verkerk, Illés J. Doszpod, Isabella Mengarelli, Tibor Magyar, Alexandra Polyák, Bence Pászti, Igor R. Efimov, Ronald Wilders, István Koncz

**Affiliations:** 1 Department of Experimental Cardiology, Heart Center, Amsterdam Cardiovascular Sciences, Amsterdam UMC, University of Amsterdam, 1105 AZ Amsterdam, The Netherlands; 2 Department of Medical Biology, Amsterdam Cardiovascular Sciences, Amsterdam UMC, University of Amsterdam, 1105 AZ Amsterdam, The Netherlands; 3 Department of Pharmacology and Pharmacotherapy, Albert Szent-Györgyi Medical School, University of Szeged, 6721 Szeged, Hungary; 4 Department of Biomedical Engineering, The George Washington University, Washington, DC 20052, USA; 5 Department of Biomedical Engineering, Northwestern University, Chicago, IL 60611, USA; 6 Department of Medicine, Northwestern University, Chicago, IL 60611, USA

**Keywords:** acetylcholine, action potential duration, Purkinje fiber, human induced pluripotent stem cell-derived cardiomyocytes (hiPSC-CMs), L-type Ca^2+^ current, repolarization, cellular electrophysiology, patch clamp recordings, computer simulations

## Abstract

Vagal nerve stimulation (VNS) holds a strong basis as a potentially effective treatment modality for chronic heart failure, which explains why a multicenter VNS study in heart failure with reduced ejection fraction is ongoing. However, more detailed information is required on the effect of acetylcholine (ACh) on repolarization in Purkinje and ventricular cardiac preparations to identify the advantages, risks, and underlying cellular mechanisms of VNS. Here, we studied the effect of ACh on the action potential (AP) of canine Purkinje fibers (PFs) and several human ventricular preparations. In addition, we characterized the effects of ACh on the L-type Ca^2+^ current (I_CaL_) and AP of human induced pluripotent stem cell-derived cardiomyocytes (hiPSC-CMs) and performed computer simulations to explain the observed effects. Using microelectrode recordings, we found a small but significant AP prolongation in canine PFs. In the human myocardium, ACh slightly prolonged the AP in the midmyocardium but resulted in minor AP shortening in subepicardial tissue. Perforated patch-clamp experiments on hiPSC-CMs demonstrated that 5 µM ACh caused an ≈15% decrease in I_CaL_ density without changes in gating properties. Using dynamic clamp, we found that under blocked K^+^ currents, 5 µM ACh resulted in an ≈23% decrease in AP duration at 90% of repolarization in hiPSC-CMs. Computer simulations using the O’Hara–Rudy human ventricular cell model revealed that the overall effect of ACh on AP duration is a tight interplay between the ACh-induced reduction in I_CaL_ and ACh-induced changes in K^+^ currents. In conclusion, ACh results in minor changes in AP repolarization and duration of canine PFs and human ventricular myocardium due to the concomitant inhibition of inward I_CaL_ and outward K^+^ currents, which limits changes in net repolarizing current and thus prevents major changes in AP repolarization.

## 1. Introduction

Patients diagnosed with heart failure have a low vagal tone and high sympathetic activity [1]. Vagal nerve stimulation (VNS) possesses a strong basis as a potentially effective treatment modality for chronic heart failure [2]. Three clinical trials have been completed, i.e., the Autonomic Neural Regulation Therapy to Enhance Myocardial Function in Heart Failure (ANTHEM-HF) trial [3], the Neural Cardiac Therapy for Heart Failure (NECTAR-HF) trial [4], and the Increase in Vagal Tone in Heart Failure (INOVATE-HF) trial [5], and a fourth trial is ongoing, i.e., the ANTHEM-Autonomic Regulation Therapy to Enhance Myocardial Function and Reduce Progression of Heart Failure with Reduced Ejection Fraction (ANTHEM-HFrEF) trial [6]. However, there are gaps in the knowledge about the effect of acetylcholine (ACh) on cardiac repolarization. For example, intracoronary administration of ACh to a patient who had normal QT interval unmasked abnormal QT interval prolongation and induced torsades de pointes (TdP) [7]. In addition, the same research group found that intracoronary administration of ACh induced prolongation of monophasic AP (MAP) duration and caused TdP in a patient in whom intravenous atropine administration did not induce any change in MAP duration [8]. In the case of both the congenital and the acquired form of long QT syndrome, malignant arrhythmias including TdP are presumably attributable to a reentrant mechanism and are conceivably precipitated or triggered by an early afterdepolarization (EAD)-elicited triggered response originating from Purkinje fibers (PFs) or from cardiomyocytes located in the midmyocardial regions of the ventricles [9,10,11]. EADs are generally associated with prolongation of repolarization and facilitated by lower heart rates [12,13].

Thus, there is a need to obtain more detailed information on the electrophysiological effects of ACh in human heart or, as an alternative, in animal hearts close to human hearts in size and electrophysiology. Over the past 40 years, different species and cardiac tissues have been used to evaluate the effects of ACh on AP repolarization, but the described effects are highly variable between species and also within a single species. For example, in cardiac PFs, APs may prolong, shorten, or stay unaltered in response to ACh (see Table 1 and Section 4.2.1 for detailed discussion). Apart from an interspecies difference in the electrophysiological effects of ACh, there is also a difference in response to ACh between PFs and ventricular tissue from the same heart [14] and between ventricular layers from the same heart [15,16]. The species-specific and tissue-specific effects of ACh point to a delicate balance of multiple ACh-sensitive inward and outward membrane currents that has both species-specific and tissue-specific characteristics.

In the present study, we characterized the effects of ACh on AP repolarization of canine PFs as well as regional effects in human tissue. We found that 5 min exposure to ACh (1 and 5 µM) lengthened AP duration (APD) at 90% of repolarization (APD_90_) in PFs from both normal (non-trained) and exercise-trained dogs and slightly prolonged APD in a human midmyocardial slice preparation. On the other hand, ACh shortened APD in human atrial and subepicardial ventricular preparations. However, the observed effects on AP repolarization in canine PFs and human ventricular tissue were relatively mild. Changes in APD can be due to changes in outward K^+^ current or inward L-type Ca^2+^ current (I_CaL_), or a combination of both. The ACh-activated K^+^ current, I_K,ACh_, is not present in human ventricular cardiomyocytes [24,25] and muscarinic cholinergic agonists may decrease the rapid and slow components of the delayed rectifier K^+^ current (I_Kr_ and I_Ks_, respectively) [26,27]. However, the effects of muscarinic cholinergic agonists on I_CaL_ in animal species remain controversial [28]. Some studies showed no effects, while others found a decrease in I_CaL_ (for details, see Section 4.3.1). Data on ACh effects on I_CaL_ in human ventricular cardiomyocytes or in human induced pluripotent stem cell derived cardiomyocytes (hiPSC-CMs) as a surrogate are lacking. Therefore, we performed perforated patch-clamp experiments on hiPSC-CMs ourselves and found a significant decrease in I_CaL_, without changes in gating properties. Using dynamic clamp, we tested the effects of ACh on APs under conditions of blocked K^+^ currents. We found that ACh resulted in AP shortening in hiPSC-CMs under such conditions. Computer simulations revealed that there is a tight interplay between the ACh-induced reduction in I_CaL_ and ACh-induced changes in I_Kr_ and I_Ks_, which limits changes in net repolarizing current and thus prevents major changes in AP repolarization.

## 2. Materials and Methods

### 2.1. Conventional Microelectrode Recordings

#### 2.1.1. Dog Heart Preparations

We used heart preparations from normal (non-trained) beagle dogs, but also from exercise-trained ones. The rationale for including the exercise-trained dogs is that physical training results in differences in parasympathetic stimulation (for review, see Zanesco and Antunes [29]) and thus may affect ACh-induced effects on AP repolarization. Dogs in the trained group were 12 months old at the beginning of the long-term endurance training protocol. Running sessions were performed on a large animal treadmill system (Dog Runner K9 Racer Treadmill, Spanadra, Dendermonde, Belgium). Trained animals underwent a 16-week-long training session. The protocol started with a one-week-long warm-up period. Thereafter, animals were trained for 5 days a week with 2 × 90 min at a speed of 12–18 km/h (increasing protocol) and with 2 × 50 min interval running at fixed speeds of 4 and 22 km/h a day for 16 weeks. The training intensity was maintained with the use of 5% to 12% inclination. The training protocol was tested in preliminary experiments and set to the maximum level that could be performed without distress.

Canine hearts were removed through a right lateral thoracotomy from anesthetized (sodium pentobarbital (60 mg/kg iv)) dogs of either sex weighing 10–15 kg. Free-running PFs were identified as false tendons and isolated from both ventricles. PFs were gently cut out with small pieces of ventricular muscle with a fine pair of scissors and these muscular parts were pinned to the rubber floor of the chamber. At impalement, PFs were observed under a surgical microscope (Zeiss OPMI PRO). The preparations were placed in Locke’s solution and made it possible to equilibrate for at least 2 h while superfused (flow rate 4–5 mL/min) also with Locke’s solution containing (in mM): NaCl 120, KCl 4, CaCl_2_ 2, MgCl_2_ 1, NaHCO_3_ 22, and glucose 11. The pH of this solution was 7.40–7.45 when gassed with 95% O_2_ and 5% CO_2_ at 37 °C. All experiments were performed at 37 °C. During the equilibration period, preparations were stimulated by electrical pulses of 1 ms in duration and twice the diastolic threshold in intensity through bipolar platinum electrodes. Transmembrane potentials were recorded using glass capillary microelectrodes filled with 3 M KCl (tip resistance: 5 to 15 MΩ). The microelectrodes were coupled through an Ag–AgCl junction to the input of a high-impedance, capacitance-neutralizing Experimetria 2011 amplifier (Experimetria Ltd., Budapest, Hungary). Intracellular recordings were displayed on a storage oscilloscope (Hitachi V-555) and led to a computer system (APES) designed for on-line determination of AP parameters. Baseline recordings were obtained after an equilibration period. ACh was purchased from Sigma/Merck.

#### 2.1.2. Human Heart Preparations

##### Human Atrial Preparation (Left Atrial Appendage)

Experiments on de-identified unsuitable donor hearts were approved by the Institutional Review Board of the George Washington University (Washington, DC, USA) and Washington Regional Transplant Community (Falls Church, VA, USA). A pectinate muscle of a left atrial appendage was prepared from a non-pathological human heart. AP recordings were made with the use of a Zeiss Stemi SV11 microscope, a Power1401 data acquisition interface and Spike2 software (Cambridge Electronic Design Ltd. (Cambridge, UK)), and an Electro 705 intracellular amplifier (World Precision Instruments). The preparation was superfused with oxygenated (95% O_2_ and 5% CO_2_ at 37 ± 0.5 °C) Tyrode’s solution containing (in mM): NaCl 129, KCl 4.7, NaH_2_PO_4_ 1.19, NaHCO_3_ 20, CaCl_2_ 1.3, MgCl_2_ 1.05, and glucose 11.1 (pH 7.4).

##### Human Left Ventricular Myocardial Slices

Human hearts that were unsuitable for transplantation were obtained from organ donors. The investigations conformed to the principles outlined in the *Declaration of Helsinki* of the World Medical Association. All experimental protocols were approved by the Scientific and Research Ethical Committee of the Medical Scientific Board at the Hungarian Ministry of Health (Budapest, Hungary) (ETT-TUKEB), under ethical approval No. 4991-0/2010-1018EKU (339/PI/010). Human cardiac tissue was stored at 4 °C in cardioplegic solution containing (in mM): NaCl 110, KCl 16, MgCl_2_ 16, NaHCO_3_ 10, and CaCl_2_ 1.2. To prepare myocardial ventricular preparations, a piece from the basal part of the left ventricle was glued with tissue adhesive directly to the top of the cutting stage of a vibratome (Vibratome 3000 PELCO 100 Vibratome Sectioning System, generous donation from Mr. Tamás Leisztinger). Tangential slices (400 µm thickness) were cut in cold (4 °C) Locke’s solution with a steel blade. The slices were placed in a preincubation chamber filled with oxygenated Locke’s solution at room temperature. The tissue was then allowed to equilibrate for at least 2 h during continuous superfusion (flow rate 4–5 mL/min) with Locke’s solution in the recording chamber. The solution was gassed with 95% O_2_ and 5% CO_2_ at 37 °C. The method for transmembrane potential recordings was similar to that for the canine PF preparations.

### 2.2. Patch Clamp Recordings from hiPSC-CMs

#### 2.2.1. hiPSC Culture and Cardiomyocytes Differentiation

The hiPSC line PGP1 (available at the Harvard Personal Genome Project [30], generated using the CytoTune-iPS 2.0 Sendai Reprogramming Kit, was cultured in the presence of both mTeSR1 Medium (STEMCELL Technologies, Vancouver, BC, Canada) and Essential 8 Medium (Gibco/Thermo Fisher Scientific, Waltham, MA, USA) (50/50 ratio) on Matrigel Matrix (Corning, NY, USA) coated 12-well plates with daily media change. For cardiomyocytes differentiation, we applied an adaptation of the protocol described by Maas et al. [31], as follows. When the hiPSC culture had reached 65–80% confluence (day 1 of differentiation), the medium was changed to RPMI 1640 medium containing 2% B27− supplement without insulin (RPMI/B27−; Gibco/Thermo Fisher Scientific, Waltham, MA, USA) and 6 µM CHIR99021 (Selleck Chemicals LLC, Houston, TX, USA). On day 2, RPMI 1640/B27− medium was added to the culture. On day 3, the medium was changed to RPMI 1640/B27− medium supplemented with 5 µM IWP4 (Stemgent, Beltsville, MD, USA). On day 5, the medium was changed again to RPMI 1640/B27− medium to remove the Wnt-signaling inhibitor. The cells were then maintained in RPMI 1640/B27− medium for 43 days with medium change every other day. Subsequently, the culture was switched to RPMI 1640 without glucose (Gibco/ThermoFisher Scientific, Waltham, MA, USA) supplemented with 500 µg/mL bovine serum albumin (Sigma-Aldrich/Merck, Kenilworth, NJ, USA) and 8 mM Na-L-lactate (Sigma-Aldrich/Merck, Kenilworth, NJ, USA) for 7 days to apply a metabolic enrichment for cardiomyocytes [32]. The culture was switched to RPMI 1640/B27− for 1 day.

#### 2.2.2. Preparation of hiPSC-CMs for Patch Clamp Recordings

The culture of hiPSC-derived cardiomyocytes was dissociated to single cells as follows. The culture was incubated for 10 min at room temperature in presence of a mix (50/50 ratio) of Hanks Balanced Salt Solution (HBSS, Gibco/Thermo Fisher Scientific, Waltham, MA, USA) without CaCl_2_ and MgCl_2_ and HBSS with CaCl_2_ and MgCl_2_ (HBSS 50/50). Cells were then incubated in TrypLE Select Enzyme (Gibco/Thermo Fisher Scientific, Waltham, MA, USA) (5X) in phosphate buffered saline (DPBS; Gibco/Thermo Fisher Scientific, Waltham, MA, USA) without CaCl_2_ and MgCl_2_ for 5 min at 37 °C; HBSS 50/50 was added to the wells to dilute the enzyme and the cell layer was mechanically removed from the surface and fractionated by gently scraping it. The cells in suspension were then collected by centrifugation; the cell pellet was resuspended in a low-Ca^2+^ HEPES-buffered Tyrode solution and incubated for 15 min at 37 °C in presence of 25 µg/mL Liberase TM Research Grade (Roche/Merck, Kenilworth, NJ, USA). This low-Ca^2+^ Tyrode solution contained (in mM): 140 NaCl, 5.4 KCl, 0.01 CaCl_2_, 1.0 MgCl_2_, 5.5 glucose, 5.0 HEPES, and 14.1 creatine; pH 7.4 (NaOH). The cells were again collected by centrifugation, resuspended and dissociated by pipetting in RPMI 1640/B27− medium and seeded (100 µL cell suspension/cover glass) on Matrigel Matrix-coated round (12 mm ø) microscope cover glasses (VWR International GmbH, Darmstadt, Germany). The dissociated cardiomyocytes were then maintained in RPMI 1640/B27− medium and analyzed 8–10 days after dissociation.

#### 2.2.3. Data Acquisition

I_CaL_ and APs were recorded from hiPSC-CMs at 36 ± 0.2 °C using an Axopatch 200B amplifier (Molecular Devices, Sunnyvale, CA, USA). We used the perforated patch-clamp methodology to prevent cell dialysis-induced I_CaL_ rundown as may occur during ruptured patch-clamp measurements [33,34]. Perforated patch-clamp recordings of I_CaL_ are not only stable for long periods [35], but also leave cytosolic composition virtually unaltered, resulting in a close-to-physiological function and modulation of I_CaL_ [36]. Voltage control, data acquisition, and analysis were realized with custom software. The extracellular bath solution was a HEPES-buffered Tyrode’s solution in which K^+^ was replaced by Cs^+^ to block K^+^ currents. It contained (in mM): NaCl 140, CsCl 5.4, CaCl_2_ 1.8, MgCl_2_ 1.0, glucose 5.5, and HEPES 5.0; pH 7.4 (NaOH). Na^+^ current was blocked by adding 1 µM TTX to the bath solution. Patch pipettes (borosilicate glass, ≈3 MΩ; Harvard Apparatus, UK) were filled with solution containing (in mM): K-gluconate 125, KCl 20, NaCl 5, amphotericin-B 0.44, and HEPES 10; pH 7.2 (KOH). Potentials were corrected for the calculated liquid junction potential [37]. Cell membrane capacitance (C_m_) was calculated by dividing the time constant of the decay of the capacitive transient after a −5 mV voltage step from −40 mV by the series resistance. I_CaL_ signals were low-pass-filtered with a cutoff of 5 kHz and digitized at 10 kHz; AP signals were filtered and digitized at 5 and 40 kHz, respectively. Series resistance was compensated for by at least 80%.

#### 2.2.4. L-Type Calcium Current Measurements

I_CaL_ density and gating properties were measured from a −70 mV holding potential using a two-pulse voltage clamp protocol with a cycle length of 4 s, as detailed in Section 3.2.1, below. The first depolarizing pulse (P1) served to activate I_CaL_; the second pulse (P2) was used to analyze the inactivation properties of I_CaL_. I_CaL_ was defined as the difference between the peak current and the steady-state current. Current density was calculated by dividing the I_CaL_ amplitude by C_m_. Voltage dependence of activation and inactivation curves were fitted with the Boltzmann function I/I_max_ = A/{1.0 + exp[(V_½_ − V)/k]}, where V_½_ is the voltage of half-maximal (in)activation and k the slope factor (in mV). The decay of I_CaL_ was fitted with the double exponential equation I/I_max_ = A_f_ × exp(−t/τ_f_) + A_s_ × exp(−t/τ_s_), where A_f_ and A_s_ are the fractions, and τ_f_ and τ_s_ the time constants, of the fast and slow components, respectively.

#### 2.2.5. Action Potential Measurements 

APs were measured with the same solutions as used for the I_CaL_ measurements, except that TTX was omitted from the bath solution. Because of the presence of Cs^+^ and the consequent blockade of K^+^ currents, we used dynamic clamp [38,39] to inject a synthetic inward rectifier K^+^ current (I_K1_) with moderate rectification, which we named “Bett current” in previous studies [40,41,42] after its current–voltage relationship defined by Bett et al. [43], in order to (a) set the resting membrane potential (RMP) at close-to-physiological values, and (b) induce AP repolarization. This approach enabled us to study the effects of ACh on AP repolarization without the interference of changes in the rapid or slow delayed rectifier K^+^ currents (I_Kr_ and I_Ks_, respectively). The amount of injected current was chosen such that the AP duration at 90% of repolarization (APD_90_) was between 250 and 300 ms in absence of ACh. APs were elicited at 1 Hz by 3 ms, ≈1.3× threshold current pulses through the patch pipette. APs were characterized by RMP, AP amplitude (APA), maximum AP upstroke velocity (V_max_), and AP duration at 20, 50, and 90% of repolarization (APD_20_, APD_50_, and APD_90_, respectively). Action potential parameters from 10 consecutive action potentials were averaged.

### 2.3. Computer Simulations

The functional effects of the ACh-induced changes in I_CaL_, I_Kr_, and I_Ks_ on human ventricular cardiomyocytes were assessed by computer simulations using the O’Hara–Rudy human ventricular cell model [44]. Similarly, the Maleckar et al. human atrial cell model [45] was used to assess the functional effects of these three currents as well as the atrial-specific I_K,ACh_ on human atrial cardiomyocytes. In either model, the effects of ACh on I_CaL_ were implemented as a 14.9% decrease in its fully activated conductance, as observed in our present study. The effects of ACh on I_Kr_ were incorporated by a 23% decrease in its fully activated conductance and a +17.3 mV shift in its steady-state activation curve, as observed in our previous study [26]. The effects of ACh on I_Ks_ were implemented as a 47% decrease in its fully activated conductance, as observed by Freeman and Kass [27]. To activate I_K,ACh_ in the Maleckar et al. human atrial cell model [45], the model ACh concentration, which affects only I_K,ACh_ in the model, was set to 1 µM.

The CellML code of either model, as available from the CellML Model Repository [46] at https://www.cellml.org/ (accessed on 21 September 2022), was edited and run in version 0.9.31.1409 of the Windows-based Cellular Open Resource (COR) environment [47]. All simulations were run for the duration of a train of 200 action potentials in order to reach steady-state behavior. Data from the final ten action potentials were used for analysis.

### 2.4. Statistics

Data are expressed as mean ± SEM. Statistical analysis was carried out with SigmaStat 3.5 software (Systat Software, Inc., San Jose, CA, USA). Paired or unpaired *t*-tests were used to compare two groups. Two-way repeated measures ANOVA followed by pairwise comparison using the Student–Newman–Keuls test was used to compare multiple groups. *p* < 0.05 was considered statistically significant.

## 3. Results

### 3.1. Standard Microelectrode Measurements

#### 3.1.1. Effect of ACh in Canine Purkinje Fibers

**Figure 1:**
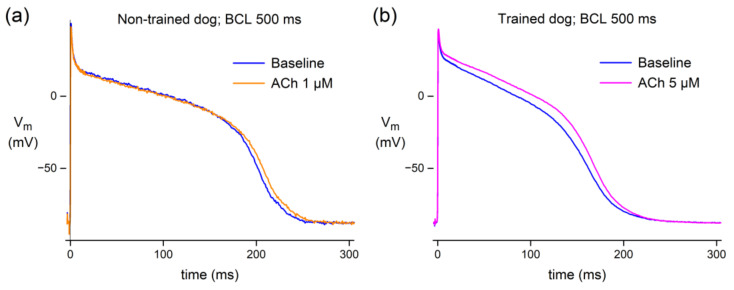
Typical action potential (AP) waveforms of canine Purkinje fibers (PFs) in absence (baseline) and presence of acetylcholine (ACh). (**a**) Effect of 5 min exposure to 1 µM ACh on the AP of a PF of a non-trained dog at a basic cycle length (BCL) of 500 ms. (**b**) Effect of 5 min exposure to 5 µM ACh on the AP of a PF of an exercise-trained dog. AP recordings of each panel originate from the same preparation and impalement. Average AP data from six non-trained and four exercise-trained dogs are listed in Table 2.

We tested the effects of ACh in PFs from six non-trained dogs. Figure 1a shows typical AP recordings at a cycle length of 500 ms in absence (baseline) and in presence of 1 µM ACh. A typical example shows a minor AP prolongation. Table 2 shows the average AP parameters of these six non-trained dogs under baseline conditions and in the presence of 1 µM ACh at a cycle length of 500 ms. ACh induced a small but statistically highly significant increase in APD_50_ and APD_90_, while other AP parameters were unaffected (Figure 1a; Table 2). These experiments demonstrate that ACh slightly prolongs the AP in PFs of non-trained dogs.

We also characterized the effects of ACh in PFs of four exercise-trained dogs at a cycle length of 500 ms. The rationale for doing so is that exercise training results in differences in parasympathetic stimulation (for review, see Zanesco and Antunes [29]), thus potentially affecting the ACh-induced effects. The PFs of exercise-trained dogs had faster AP upstrokes, larger AP amplitudes, and shorter APDs than non-trained dogs in absence of ACh (Table 2, baseline conditions). Ionic and intracellular Ca^2+^ homeostasis remodeling in response to exercise training is well known for ventricular cardiomyocytes (see Kemi et al. [48], Wang and Fitts [49], and Kui et al. [50], and primary references cited therein). However, although interesting, the underlying mechanism of these differences in AP parameters was not the topic of the present study and was therefore not further explored. In PFs from the exercise-trained dogs, we tested the effects of 5 µM ACh at a cycle length of 500 ms. ACh significantly prolonged APD_25_, APD_50_, and APD_90_ (Figure 1b; Table 2). In addition, the AP amplitude showed a slight but statistically significant increase. As found in non-trained dogs, albeit at 1 µM ACh instead of 5 µM ACh, the AP prolongation was relatively mild.

#### 3.1.2. Effect of ACh in Human Atrial and Ventricular Preparations

**Figure 2:**
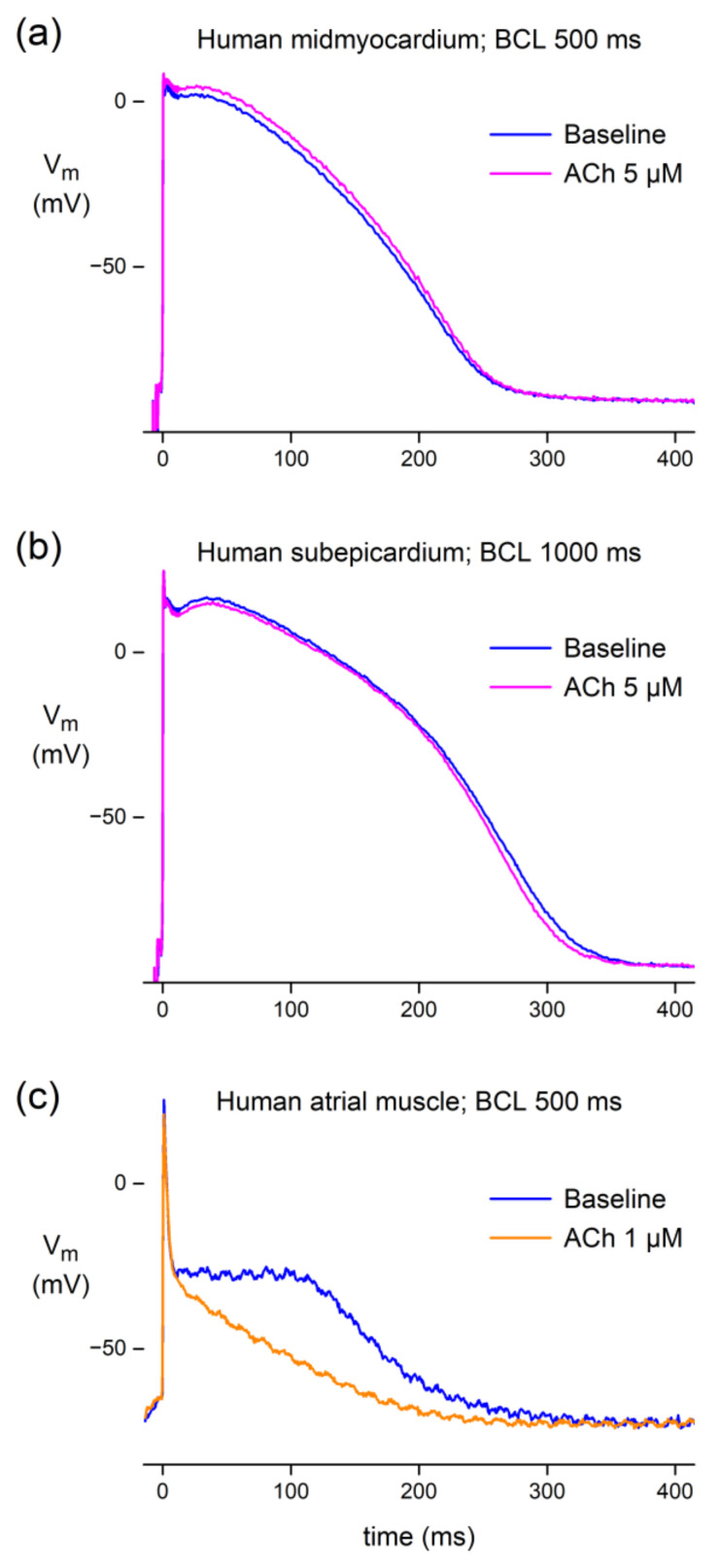
AP recordings obtained with microelectrode impalements of human ventricular and atrial myocardial tissue from three undiseased donor hearts. (**a**) Midmyocardial AP recording under baseline conditions and upon 5 min exposure to 5 μM ACh at a basic cycle length (BCL) of 500 ms. (**b**) Subepicardial AP recording under baseline conditions and upon 10 min exposure to 5 μM ACh at a BCL of 1000 ms. (**c**) Atrial AP recording under baseline conditions and upon 5 min exposure to 1 μM ACh at a BCL of 500 ms. AP recordings of each panel originate from the same preparation and impalement.

Having established that ACh may result in a small AP prolongation in PFs of dogs, independent of the training status of the dogs, we tested the effects of ACh on ventricular and atrial preparations that we could prepare from a total of five undiseased human donor hearts. Recordings from three preparations (two ventricular, one atrial) obtained from three different hearts were successful. Microelectrode impalement of a human midmyocardial cell in a heart slice from an undiseased donor heart demonstrated that ACh (5 µM) raised the AP plateau and slightly prolonged the AP at a basic cycle length of 500 ms (Figure 2a). However, in a human subepicardial cell impaled in a heart slice from another undiseased donor heart, ACh (5 μM) depressed the AP plateau and slightly abbreviated the APD at a basic cycle length of 1000 ms (Figure 2b). In an atrial preparation, i.e., a pectinate muscle of a left atrial appendage from a third non-pathological human heart, ACh (1 µM) strongly depressed the AP plateau and elicited a marked abbreviation of the APD at a basic cycle length of 500 ms (Figure 2c). Thus, ACh may result in either a small AP prolongation or shortening in human ventricular tissue, depending on the location of the microelectrode impalement, whereas the atrial AP may be markedly shortened.

### 3.2. Patch Clamp Measurements

#### 3.2.1. Effects of ACh on I_CaL_ Density

**Figure 3:**
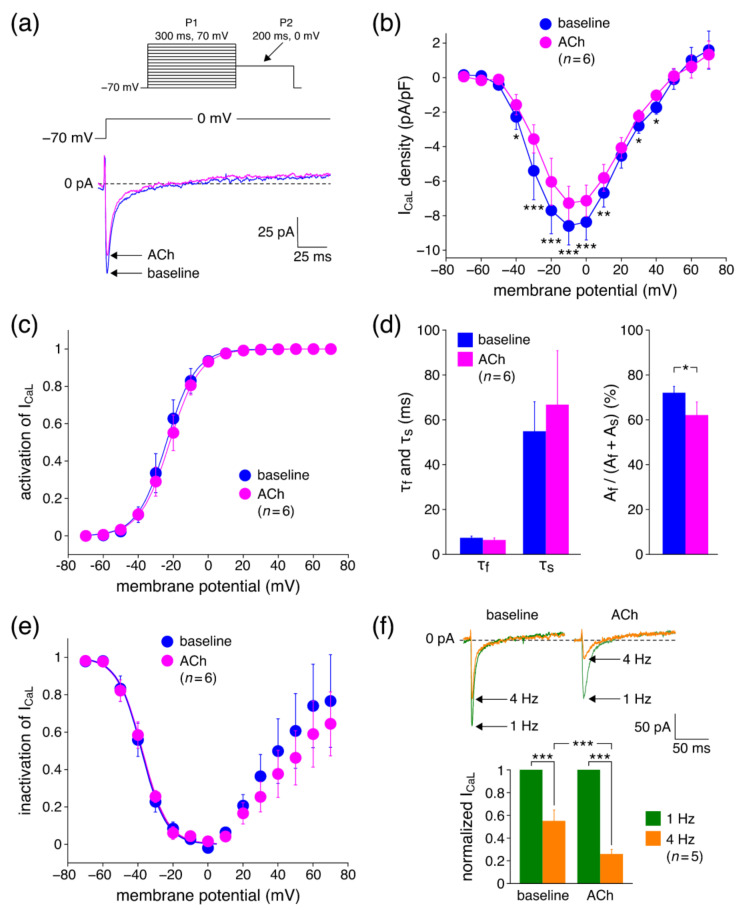
Effects of ACh on L-type Ca^2+^ current (I_CaL_) in human induced pluripotent stem cell derived cardiomyocytes (hiPSC-CMs). (**a**) Typical example of I_CaL_ in absence (baseline) and presence of ACh (blue and magenta traces, respectively). Inset: double pulse protocol used to measure I_CaL_. (**b**) Average current–voltage relationship of I_CaL_ (*n* = 6). * *p* < 0.05; ** *p* < 0.01; *** *p* < 0.001 (two-way repeated measures ANOVA). (**c**) Voltage dependence of activation of I_CaL_ (*n* = 6). (**d**) Rate of I_CaL_ inactivation in response to depolarizing pulses from −70 to 0 mV (with fast and slow time constants τ_f_ and τ_s_, respectively; left) and relative amplitude of its fast and slow components (right) (*n* = 6). * *p* < 0.05 (paired *t*-test). (**e**) Voltage dependence of inactivation of I_CaL_ (*n* = 6). (**f**) I_CaL_ measured at 1 and 4 Hz in response to 200 ms depolarizing pulses from −70 to 0 mV (*n* = 5). *** *p* < 0.001 (two-way repeated measures ANOVA).

Our microelectrode recordings demonstrated that ACh may result in either a slight AP prolongation or a slight AP shortening, depending on the location of the impalement. These changes contradict the ACh-induced decrease in both I_Kr_ and I_Ks_ from which we expect a substantial AP prolongation [26,51]. However, ACh may potentially also decrease I_CaL_, which would have an AP shortening effect. Therefore, we next carried out voltage clamp experiments on hiPSC-CMs to determine I_CaL_ properties in absence (baseline) and presence of 5 µM ACh. Figure 3a shows typical traces of I_CaL_ upon depolarizing steps from −70 to 0 mV. ACh significantly decreased the I_CaL_ amplitude, which is also evident from the average current–voltage (I-V) relationships of Figure 3b. For example, at 0 mV, I_CaL_ density was reduced by 14.9 ± 1.6% (*n* = 6) from −8.4 ± 1.0 (baseline) to −7.1 ± 0.8 (ACh) pA/pF. To determine the voltage dependence of activation of I_CaL_, the I-V relationships of each individual cell under baseline conditions and in the presence of ACh were corrected for driving force, normalized to maximum peak current, and fitted to a Boltzmann curve, characterized by its voltage of half-activation V_½_ and its slope factor k. Fitting the average activation data in absence and presence of ACh yielded Boltzmann curves that were virtually overlapping (Figure 3c), indicating unaltered activation. This was substantiated by the average V_½_ and k values obtained under baseline conditions and in the presence of ACh. V_½_ averaged −22.4 ± 2.6 and −21.5 ± 2.5 mV (*p* = 0.53; paired *t*-test) and k averaged 7.1 ± 0.9 and 7.6 ± 0.9 mV (*p* = 0.69; paired *t*-test) under baseline conditions and in the presence of ACh, respectively.

The decay of I_CaL_ at 0 mV was fitted with a double exponential function to determine the rate of I_CaL_ inactivation and the relative amplitude of its fast and slow components. Neither the fast (τ_f_) nor the slow (τ_s_) time constant of inactivation were significantly affected by ACh (Figure 3d, left). The amplitude of the slow inactivating component (A_s_) was not significantly affected, but the amplitude of the fast inactivating component (A_f_) was significantly reduced, resulting in a reduced role of the fast inactivation in the total inactivation of I_CaL_ (Figure 3d, right). The voltage dependence of inactivation of I_CaL_ was assessed using a 300 ms P1 prepulse followed by the 200 ms P2 test pulse to 0 mV (Figure 3a, inset). The resulting inactivation curves, normalized to the largest I_CaL_, are shown in Figure 3e. The V_½_ of inactivation averaged −38.5 ± 3.1 and −39.2 ± 3.4 mV (*p* = 0.55; paired *t*-test) and k −6.4 ± 0.7 and −7.0 ± 0.9 mV (*p* = 0.21; paired *t*-test) in absence and presence of ACh, respectively, indicating that the voltage dependence of inactivation was not affected by ACh. Figure 3e shows that the steady-state inactivation curve (or availability curve) rises positive to 0 mV, which is a well-known feature for I_CaL_ [52]. However, this ‘relief’ from inactivation was not significantly different between baseline and the presence of ACh. Next, we tested whether the effects of ACh on I_CaL_ are frequency dependent by applying a 200 ms depolarizing pulse from −70 to 0 mV at 1 and 4 Hz in absence and presence of ACh. Both under baseline and ACh conditions, the I_CaL_ amplitude measured at 4 Hz was substantially decreased as compared to the current measured at 1 Hz (Figure 3f), consistent with the slow recovery from inactivation of I_CaL_ [52]. However, in presence of ACh, this reduction was significantly larger as compared to baseline conditions (74.0 ± 3.7% (ACh) vs. 45.0 ± 8.8% (baseline); *n* = 5).

#### 3.2.2. Effects of ACh on APs with Limited Interference of K^+^ Currents

**Figure 4:**
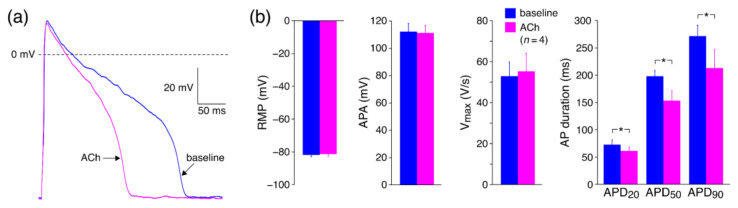
Effects of ACh on APs of human induced pluripotent stem cell derived cardiomyocytes (hiPSC-CMs) with limited interference of K^+^ currents. (**a**) Typical examples of APs elicited at 1 Hz in absence (baseline) and presence of ACh. (**b**) Average AP parameters in absence and presence of ACh (*n* = 4). RMP: resting membrane potential; APA: AP amplitude; V_max_: maximum AP upstroke velocity; APD_20_, APD_50_, and APD_90_: AP duration at 25, 50, and 90% repolarization, respectively. * *p* < 0.05 (paired *t*-test).

In a final series of patch clamp experiments, we tested the effects of ACh on APs of hiPSC-CMs without potential interference of I_Kr_ and I_Ks_. Therefore, we blocked K^+^ currents with Cs^+^ and induced AP repolarization using dynamic clamp to inject a repolarizing current that was voltage dependent but not ACh dependent. This allowed us to investigate the effects of ACh-induced changes in I_CaL_ per se on APs. Figure 4a shows a typical example. Figure 4b shows the average effects of ACh on the AP parameters of four hiPSC-CMs. ACh resulted in AP shortening, which is evident from the significant decrease in APD_20_, APD_50_, and APD_90_. For example, APD_90_ decreased from 271 ± 18 to 213 ± 30 ms, which was a 22.6 ± 6.6% decrease. In addition, ACh lowered the AP plateau and tended to decrease the APA, although this effect did not reach the level of significance. RMP and V_max_ were unaltered in response to ACh. Thus, these experiments demonstrate that ACh results in AP shortening in absence of ACh-induced changes in I_Kr_ and I_Ks_.

### 3.3. In Silico Experiments

#### 3.3.1. Human Ventricular Cell Model

In canine and human ventricular myocardium, we found minor ACh-induced changes in APD (Figure 1 and Figure 2), despite the ACh-induced reduction of I_CaL_ (Figure 3). However, a large AP shortening was observed when K^+^ currents were blocked (Figure 4). This suggests that a simultaneous ACh-induced decrease in one or more K^+^ currents, as observed for both I_Kr_ and I_Ks_ [26,51], limits ACh-induced changes in APD. This hypothesis was tested in a series of in silico experiments, using the comprehensive O’Hara–Rudy human ventricular cell model [44] at a stimulus frequency of 1 Hz, as a realistic frequency for human cardiomyocytes under vagal tone.

**Figure 5:**
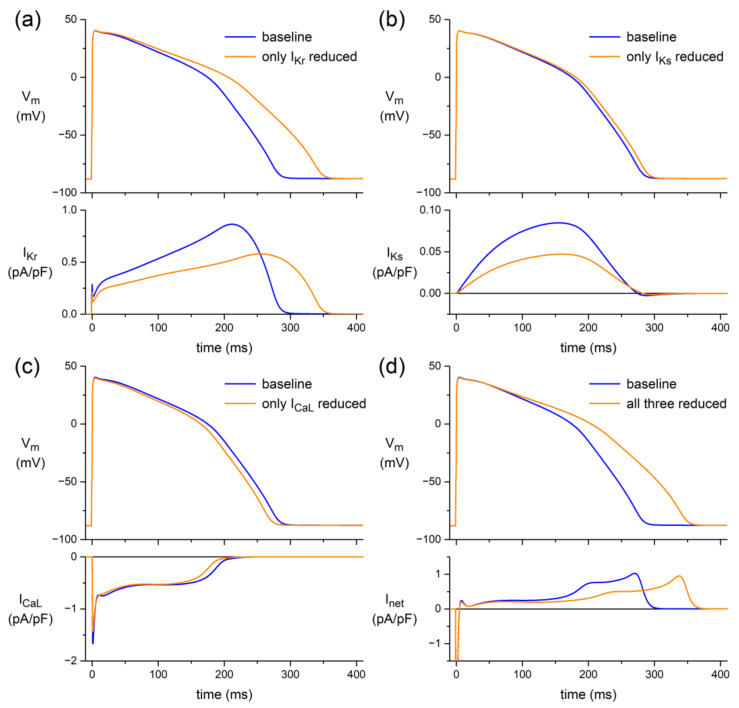
Effects of ACh-induced changes in the L-type Ca^2+^ current (I_CaL_) and the rapid and slow delayed rectifier K^+^ currents (I_Kr_ and I_Ks_, respectively) on the electrical activity of the O’Hara–Rudy human ventricular cell model [44] at 1 Hz. (**a**) Membrane potential (V_m_; top) and associated I_Kr_ (bottom) if ACh affects only I_Kr_. (**b**) V_m_ (top) and associated I_Ks_ (bottom) if ACh affects only I_Ks_. (**c**) V_m_ (top) and associated I_CaL_ (bottom) if ACh affects only I_CaL_. (**d**) V_m_ (top) and associated net membrane current (I_net_; bottom) if ACh affects all three currents, i.e., I_CaL_, I_Kr_, and I_Ks_. Note differences in current scales.

First, we limited the simulated effects of ACh to I_Kr_, applying the 23% decrease in its fully activated conductance (density) and the +17.3 mV shift in its steady-state activation curve that we observed in the voltage clamp experiments on hiPSC-CMs of our previous study [26]. The resulting decrease in I_Kr_ led to an increase in APD_90_ of as much as 64 ms (+24%; Figure 5a). Similarly, we tested the effects of an ACh-induced reduction in I_Ks_ per se, reducing the I_Ks_ fully activated conductance by 47%, as observed by Freeman and Kass [27]. Because I_Ks_ is about one order of magnitude smaller than I_Kr_, the resulting increase in APD_90_ is only 7.8 ms (+2.9%; Figure 5b). An ACh-induced reduction in both I_Kr_ and I_Ks_ resulted in an increase in APD_90_ of 79 ms (+29%).

To test the effects of an ACh-induced reduction in I_CaL_ per se, we reduced its fully activated conductance by 14.9%, as observed in our present study. APD_90_ was decreased by 11.1 ms (−4.1%; Figure 5c). This AP shortening effect was not sufficient to completely resist the combined AP prolonging effects of I_Kr_ and I_Ks_, as illustrated in Figure 5d. Incorporating the ACh-induced effects on all three currents in the model resulted in an AP prolongation of 66 ms (+24%). Thus, the AP shortening effect of the ACh-induced reduction in I_CaL_ per se was not sufficient to fully abolish the aforementioned combined AP prolonging effects of I_Kr_ and I_Ks_ on APD_90_ of 79 ms (+29%).

#### 3.3.2. Human Atrial Cell Model

**Figure 6:**
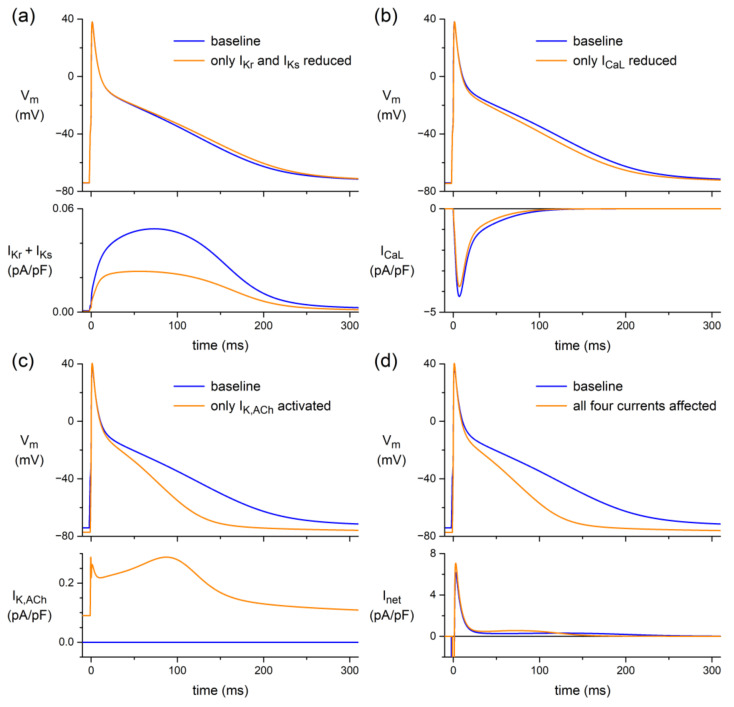
Effects of ACh-induced changes in the L-type Ca^2+^ current (I_CaL_), the rapid and slow delayed rectifier K^+^ currents (I_Kr_ and I_Ks_, respectively), and the ACh-activated K^+^ current (I_K,ACh_) on the electrical activity of the Maleckar et al. human atrial cell model [45] at 1 Hz. (**a**) Membrane potential (V_m_; top) and associated outward current carried by I_Kr_ and I_Ks_ (I_Kr_ + I_Ks_; bottom) if ACh affects only I_Kr_ and I_Ks_. (**b**) V_m_ (top) and associated inward current carried by I_CaL_ (bottom) if ACh affects only I_CaL_. (**c**) V_m_ (top) and associated ACh-activated K^+^ current (I_K,ACh_; bottom) if ACh affects only I_K,ACh_. (**d**) V_m_ (top) and associated net membrane current (I_net_; bottom) if ACh affects all four currents, i.e., I_CaL_, I_Kr_, I_Ks_, and I_K,ACh_. Note differences in current scales.

Next, identical changes in I_CaL_, I_Kr_, and I_Ks_ were applied to the comprehensive Maleckar et al. human atrial cell model [45], also at a stimulus frequency of 1 Hz. The latter model, which is also known as the “human atrial myocyte with new repolarization” (hAMr) model, was selected because it includes well-validated equations for I_K,ACh_ [53], thus allowing us to test the additional effect of the atrial-specific I_K,ACh_ on the repolarization of human atrial tissue under vagal tone. Figure 6 shows the thus-obtained results.

As illustrated in Figure 6a, the ACh-induced effect on the atrial AP is limited to a small rise of its plateau if ACh affects only I_Kr_ and I_Ks_. Although the total outward current carried by I_Kr_ and I_Ks_ is approximately halved (Figure 6a, bottom), this only small rise of the AP plateau may not be very surprising, because the total current carried by I_Kr_ and I_Ks_ is much smaller than even the separate I_Kr_ or I_Ks_ of the ventricular cell model (Figure 5a,b, bottom panels). Similarly, the effect of the small reduction in I_CaL_ is limited to a slight depression of the AP plateau (Figure 6b). In either case, the APD is only marginally affected (Figure 6a,b, top). Thus, any ACh effect on the atrial AP cannot be attributed to ACh-induced changes in I_CaL_, I_Kr_, or I_Ks_. However, activation of the atrial-specific I_K,ACh_ can strongly depress the AP plateau and seriously reduce the APD (Figure 6c, top). This may be somewhat surprising, because I_K,ACh_ is a fairly small current (Figure 6c, bottom). However, together with the small ACh-induced effects on I_CaL_, I_Kr_, and I_Ks_, its activation is able to approximately double the small outward current that flows during the AP plateau and thereby substantially shorten the AP, as illustrated in Figure 6d, in which APD_90_ is decreased by 40% from 203 to 121 ms.

## 4. Discussion

### 4.1. Overview

Here, we studied the effects of ACh on APs from a variety of canine and human cardiac tissues. Using standard microelectrode measurements, we found a small but significant APD_90_ increase in PFs from both normal (non-trained) and exercise-trained dogs (Figure 1; Table 2), and a slightly prolonged AP in a human midmyocardial ventricular preparation (Figure 2a). However, ACh shortened the APD in a human subepicardial ventricular and a human atrial preparation (Figure 2b,c). Perforated patch-clamp experiments demonstrated that ACh causes an ≈15% decrease in I_CaL_ density in hiPSC-CMs without changes in I_CaL_ gating properties, apart from a more prominent slow recovery from inactivation in the presence of ACh (Figure 3). In addition, under conditions of blocked K^+^ currents, ACh resulted in an ≈23% decrease in APD_90_ in hiPSC-CMs (Figure 4). Computer simulations using the O’Hara–Rudy human ventricular cell model [44] revealed that the overall effect of ACh on APD is a tight interplay between the ACh-induced reduction in I_CaL_ and the ACh-induced changes in I_Kr_ and I_Ks_ (Figure 5), whereas computer simulations using the Maleckar et al. human atrial cell model [45] demonstrated that I_K,ACh_ is the main determinant of ACh effects on APD in atrial cells (Figure 6).

### 4.2. Standard Microelectrode Measurements

#### 4.2.1. Canine Purkinje Fibers

We found a small but significant AP prolongation in PFs of both normal (non-trained) and exercise-trained dogs in response to ACh (Figure 1; Table 2). In our initial series of experiments on canine PFs, which were from our trained dogs, we used an ACh concentration of 5 μM. However, we then realized that a somewhat lower concentration might be physiologically more relevant, because the effect of low micromolar ACh perfusion (1 μM) seems to correlate with the effect of vagal nerve stimulation, as was demonstrated at least on heart rate by Mantravadi et al. [54]. This made us decide to use an ACh concentration of 1 μM in our subsequent series of experiments on canine PFs, which were from non-trained dogs. To allow a direct comparison between the AP parameters of our trained and our non-trained dogs, which were obtained at 5 and 1 μM ACh, respectively, we also carried out experiments on PFs from four non-trained dogs at 5 μM ACh. However, because the quality of the thus-obtained data did not meet our standards, losing the impalement during the experiment (requiring rapid re-impalement), we did not include these data, although we also observed a small but statistically significant increase in APD.

AP prolongation in response to ACh is a common finding in PFs of sheep [17,20], cat [17], and man [26]. However, APD is unaltered in PFs from cow [17] or even shortened in PFs from rabbit [17,18] and ferret [19]. Thus, the effects of ACh on APD of PFs have a clear species dependence, as can be expected from the distinct electrophysiological properties in animal species [55,56]. Additionally, within one species, data of ACh effects can be less consistent. In canine PFs, we found a small AP prolongation, but an AP shortening [21] or unaltered APD [14] have also been reported in PFs of dogs. In addition, Gilmour and Zipes [23] found an increase in APD_50_, whereas APD_90_ was not altered. Furthermore, Bailey et al. [22] reported that ACh per se had no effect on APD of canine PFs, although it significantly blunted the AP shortening effect of isoproterenol. The exact reason for these different findings in canine PFs is speculative, but we cannot exclude that it is related to the use of mongrel dogs, with mixed-breed differences between studies. The differences between species and the variable outcome within one species support the need for human experiments. 

#### 4.2.2. Human Tissue

Our human data are limited to AP recordings from three preparations from three different undiseased donor hearts that could not be used for transplantation. The recordings are from three different locations. The two ventricular preparations (one midmyocardial and one subepicardial) were exposed to 5 µM ACh, whereas 1 µM was used for the atrial preparation, because we expected a relatively high sensitivity of the atrium to ACh [57,58,59,60]. We feel that our human data are of interest, even if just descriptive. 

In human atrium, we found a severe AP shortening in response to ACh (Figure 2c). This is in agreement with findings of muscarinic receptor activation in human atrial tissue [57,58] and freshly isolated human atrial cardiomyocytes [59]. The AP shortening in human atria is importantly due to at least the activation of the ACh-activated K^+^ current (I_K,ACh_) [56,59,60] (see also Section 4.4, below).

In human midmyocardium, we found a slight APD prolongation upon exposure to ACh (Figure 2a). This is in agreement with our previous observations using optical imaging and microelectrode impalements in left ventricular tissue slices of non-pathological human hearts and in hiPSC-CMs [26]. However, it has also been reported that ACh does not have effects on APs of human ventricular papillary muscle [61]. Interestingly, in a subepicardial recording, we found a small AP shortening (Figure 2b). Regional differences in ACh effects have also been found within canine ventricle, with unaltered subendocardial APs but severely shortened subepicardial APs due to loss of the AP dome [14,15,16]. The mechanism of regional differences in response to ACh is not yet clear. Litovsky and Antzelevitch [15] mentioned that the ACh-induced subepicardial AP shortening was abolished by blockade of the transient outward K^+^ current (I_to_), which, however, was not confirmed by Yang et al. [16]. They found that I_to_ itself did not contribute to the ACh-induced AP shortening because I_to_ was not affected by ACh [16]. However, they observed a regional activation of an ACh-induced outward K^+^ current in subepicardial cardiomyocytes, which may predispose to depression or even complete loss of the dome. Independent of the mechanisms, these experiments demonstrate that ACh may increase the preexisting heterogeneity in AP morphology and duration, contributing to a pro-arrhythmogenic substrate [62,63,64,65].

### 4.3. Patch Clamp Measurements

#### 4.3.1. Effects of ACh on I_CaL_ Density

Patch clamp experiments in hiPSC-CMs demonstrated a reduction of I_CaL_ density in response to ACh (Figure 3). A reduced I_CaL_ results in a strong AP shortening in hiPSC-CMs [66]. Our ACh-induced reduction in I_CaL_ is in agreement with findings in sinoatrial node pacemaker cells of rabbit [67], atrial cardiomyocytes of cat [68], guinea pig [69], dog [14] and man [28], Purkinje fibers of sheep [70], papillary muscle of guinea pig [71], and ventricular cardiomyocytes of guinea pig [72] and dog [14,16]. On the other hand, it has also been demonstrated in various studies that ACh does not affect basal I_CaL_ activity and these studies were mostly performed in myocytes from frog, ferret, and rat [19,28,73,74]. In this regard, it should be mentioned that the effects of ACh in frog are not consistent and that an I_CaL_ reduction in bullfrog atrium has also been mentioned [75,76]. These various findings of ACh effects on I_CaL_ are thought to reflect species differences in basal activities of β-adrenergic receptors, adenylyl cyclase, phosphodiesterases, and phosphatases [28]. Thus, extrapolation of findings in animal studies to human conditions is not straightforward, indicating the need for inclusion of human tissue as in the present study.

In our I_CaL_ study, we used 5 µM ACh, but from animal studies it is known that the decreasing effects on I_CaL_ can already start at lower concentrations [67,68,71]. For example, the EC_50_ for ACh-induced inhibition of I_CaL_ is as low as 1.9 nM in cat and the reduction is maximal at around 0.1 µM and at higher concentrations [68]. We found a decrease of 14.9 ± 1.6% (with 5 µM ACh), which is largely in the same range as found in cat (19 ± 2% (1 µM)) [68] and guinea pig (25.9 ± 5.0% (10 µM)) atrial myocytes [69], sheep Purkinje fibers (25.0 ± 5.7% (1 µM)) [70], and canine ventricular myocytes (8% (0.1 µM)) [16], but it is substantially lower than reported for guinea pig papillary muscle (60.2 ± 6.3% (2 µM)) [71], and rabbit sinoatrial node cells (44.3 ± 6.1% (1 µM)) [67]. The exact reason for these differences in the ACh-induced decrease in I_CaL_ is not known, but Yang et al. [16] demonstrated that the experimentally observed decrease in I_CaL_ may depend on settings in the experimental protocol, such as the holding potential. Using a holding potential of −40 mV, the decrease in I_CaL_ was 48.6 ± 2.0%, but using a more physiological holding potential of −80 mV resulted in a decrease in only 8.2 ± 0.8%. In our study, we used a holding potential of −70 mV, which is just slightly depolarized as compared to the RMP we measured in canine and human tissue (Figure 1 and Figure 2; Table 2). Therefore, it is unlikely that the actual decrease in I_CaL_ is very different from that measured during our voltage clamp protocols.

#### 4.3.2. Effects of ACh on I_CaL_ Gating Properties

Although I_CaL_ is essential for various cardiac electrophysiological properties, arrhythmias, and ion homeostasis, we limited our study to the relation between ACh-induced changes in I_CaL_ and AP repolarization. We found that the speed of I_CaL_ inactivation was not affected by ACh, which is in agreement with findings in guinea pig atrial cardiomyocytes [69] and guinea pig papillary muscle [71]. A slowing of the I_CaL_ time course in response to ACh was observed in sheep Purkinje fibers, but this was not analyzed in detail [70]. We found that the ACh-induced reduction of I_CaL_ was more pronounced at fast pacing rates, consistent with findings in canine cardiomyocytes [14]. Likely, this is due to a reduction of recovery from inactivation in response to ACh [72]. We found no changes in voltage dependence of activation and inactivation, consistent with findings in bullfrog atrium [76]. In other studies, the shape of the I-V curves was also not drastically changed in response to ACh, suggesting that absence of changes in voltage dependence of activation is a common finding.

#### 4.3.3. Effects of ACh on APs with Limited Interference of K^+^ Currents

Using dynamic clamp to inject an ACh-independent repolarizing current, we were able to study the effects of ACh in hiPSC-CMs under conditions of blocked native K^+^ currents, which would normally result in failure of AP repolarization. We found that under such conditions, the addition of ACh resulted in a substantial AP shortening, consistent with the effects of I_CaL_ reduction on hiPSC-CM APs reported by Eroglu et al. [66]. However, despite the ACh-induced decrease in I_CaL_, we observed an AP prolongation in hiPSC-CMs in response to ACh in our previous study [26] rather than an AP shortening. This indicates that ACh-induced changes in K^+^ currents normally prevent major AP shortening.

### 4.4. In Silico Experiments

Our in silico experiments using the comprehensive O’Hara–Rudy human ventricular cell model [44] confirmed our hypothesis that the effects of the ACh-induced reduction of I_CaL_ on APD are limited by the concomitant decrease in I_Kr_ and I_Ks_. Actually, the combined AP prolonging effects of the decrease in the outward I_Kr_ and I_Ks_ are stronger than the AP shortening effects of the reduction of the inward I_CaL_, so that the net effect is an AP prolongation in response to ACh (Figure 5). However, we cannot exclude that we underestimated the actual ACh-induced reduction of I_CaL_ in adult human ventricular myocytes due to the immature Ca^2+^ handling of our hiPSC-CMs [77,78,79]. In our voltage clamp experiments on hiPSC-CMs, I_CaL_ density decreased by only ≈15% (Figure 3), whereas Bett et al. [80] reported an ≈35% decrease in ferret right ventricular myocytes, Liang et al. [81] reported an ≈37% decrease in murine embryonic cardiomyocytes, independent of their developmental stage, and Calloe et al. [14] reported an ≈31% decrease in canine left ventricular myocytes. In light of the potential interference of the holding potential (see Section 4.3.1 above), it is important to note that Bett et al. [80] and Calloe et al. [14] used a holding potential of −70 mV and −80 mV, respectively, whereas Liang et al. [81] used a holding potential of −40 mV.

Our simulations using the comprehensive Maleckar et al. human atrial cell model [45] confirmed the aforementioned experimental observations that the human atrial AP is substantially shortened in response to ACh and that this shortening is importantly due to activation of I_K,ACh_ (see Section 4.2.2 above). In our simulations, we also observed a hyperpolarization by ≈3 mV in response to activation of I_K,ACh_ (Figure 6c,d, top). This slight hyperpolarization is in line with experimental findings [57,59] and previous simulations, e.g., by Bayer et al. [57], who used a modified version of the Courtemanche–Ramirez–Nattel human atrial cell model [82].

## 5. Conclusions

ACh results in minor changes in AP repolarization and duration in canine PFs and human ventricular myocardium due to the concomitant inhibition of inward calcium current (I_CaL_) and outward K^+^ currents (I_Kr_ and I_Ks_), which limits changes in net repolarizing current and thus prevents major changes in AP repolarization

## Figures and Tables

**Table 1:** Effects of acetylcholine on action potential duration of Purkinje fibers in animal studies.

Species	APD	Reference
Rabbit	↓	Carmeliet and Ramon [17]
	↓	Mubagwa and Carmeliet [18]
Ferret	↓	Boyett et al. [19]
Cow	↔	Carmeliet and Ramon [17]
Sheep	↑	Lipsius and Gibbons [20]
	↑	Carmeliet and Ramon [17]
Cat	↑	Carmeliet and Ramon [17]
Dog	↓	Gadsby et al. [21]
	↔	Bailey et al. [22]
	↔	Gilmour and Zipes [23]
	↔	Calloe et al. [14]

APD: action potential duration; ↓: decrease; ↑: increase; ↔, unaltered.

**Table 2:** Effects of acetylcholine (ACh) on action potential characteristics of canine Purkinje fibers at a cycle length of 500 ms.

	Non-Trained Dogs (*n* = 6)		Trained Dogs (*n* = 4)	
	Baseline	1 µM ACh	Baseline	5 µM ACh
RMP (mV)	−85.3 ± 0.7	−85.7 ± 1.7	−84.6 ± 1.9	−84.6 ± 1.7
APA (mV)	118.5 ± 5.5	117.9 ± 4.8	135.2 ± 3.0 ^#^	138.1 ± 2.4 *
V_max_ (V/s)	272.2 ± 54.8	268.6 ± 51.5	486.6 ± 51.6 ^#^	514.1 ± 65.4
APD_25_ (ms)	70.1 ± 26.4	72.0 ± 26.1	35.0 ± 5.4	45.4 ± 7.3 *
APD_50_ (ms)	197.3 ± 10.9	202.3 ± 11.2 ***	147.3 ± 9.3 ^#^	158.5 ± 10.1 *
APD_90_ (ms)	248.9 ± 11.7	254.1 ± 11.7 ***	203.5 ± 12.9 ^#^	209.0 ± 13.4 *

Data are mean ± SEM. RMP: resting membrane potential; APA: action potential (AP) amplitude; V_max_: maximum AP upstroke velocity; APD_25_, APD_50_, and APD_90_: AP duration at 25, 50, and 90% repolarization, respectively. * *p* < 0.05 ACh vs. baseline (paired *t*-test). *** *p* < 0.001 ACh vs. baseline (paired *t*-test). ^#^ *p* < 0.05 trained vs. non-trained (unpaired *t*-test).

## Data Availability

Data are available to academic researchers upon request.
